# Development, piloting, and evaluation of an evidence-based informed consent form for total knee arthroplasty (EvAb-Pilot): a protocol for a mixed methods study

**DOI:** 10.1186/s40814-021-00843-x

**Published:** 2021-05-13

**Authors:** Alina Weise, Julia Lühnen, Stefanie Bühn, Felicia Steffen, Sandro Zacher, Julia Lauberger, Deha Murat Ates, Andreas Böhmer, Henning Rosenau, Anke Steckelberg, Tim Mathes

**Affiliations:** 1 Institute for Research in Operative Medicine, Faculty of Health–School of Medicine, University of Witten/Herdecke, Ostmerheimer Str. 200, Building 38, 51109 Cologne, Germany; 2 Institute for Health and Nursing Science, Medical Faculty, Martin Luther University Halle-Wittenberg, Magdeburger Straße 8, 06112 Halle (Saale), Germany; 3 Department for Criminal Law, Law of Criminal Procedure and Medical Law, Faculty of Law, Economics and Business, Martin Luther University Halle-Wittenberg, Universitätsplatz 6, 06108 Halle (Saale), Germany; 4 Department of Trauma and Orthopedic Surgery, University of Witten/Herdecke, Cologne-Merheim Medical Center, Ostmerheimer Str. 200, 51109 Cologne, Germany; 5 Department of Anaesthesiology and Intensive Care Medicine, University of Witten-Herdecke, Cologne-Merheim Medical Center, Ostmerheimer Straße 200, 51109 Cologne, Germany

**Keywords:** Informed consent, Informed consent form, Risk communication, Evidence-based health information, Nocebo effect, Anxiety, Total knee arthroplasty (TKA), Pilot, Mixed methods

## Abstract

**Background:**

Practitioners frequently use informed consent forms to support the physician-patient communication and the informed consent process. Informed consent for surgery often focuses on risk centered information due to high liability risks for treatment errors. This may affect patients’ anxiety of adverse events and the nocebo effect. This study focuses on the optimization of pre-surgical information on risks and complications, and at the same time reconciles these information with legal requirements.

**Methods:**

The development, piloting, and evaluation of evidence-based informed consent forms for total knee arthroplasty (TKA) and related anesthesia procedures will follow the UK MRC Framework for developing and evaluating complex interventions. Conducting different sub-studies, we will (I) qualitatively explore the information acquisition and decision-making processes, (II) develop and pilot test evidence-based informed consent forms on the example of TKA and related anesthesia procedures, (III) conduct a monocentric interrupted time series (ITS) pilot study to evaluate the effects of evidence-based informed consent forms in comparison with standard consent forms, and (IV) perform a process evaluation to identify barriers and facilitators to the implementation of the intervention and to analyze mechanisms of impact.

**Discussion:**

The evidence-based and understandable presentation of risks in informed consent forms aims at avoiding distorted risk depiction and strengthening the patients’ competencies to correctly assess the risks of undergoing surgery. This might reduce negative expectations and anxiety of adverse events, which in turn might reduce the nocebo effect. At the same time, the practitioners’ acceptance of evidence-based informed consent forms meeting legal requirements could be increased.

**Trial registration:**

ClinicalTrials.gov, NCT04669483. Registered 15 December 2020.

German Clinical Trials Registry, DRKS00022571. Registered 15 December 2020

## Introduction

### Background and rationale

In most countries, every medical intervention requires informed consent [1–4]. Appropriate informed consent procedures may include the presentation of comprehensible information about the necessity and kind of the intervention, mechanism of action, material risks, and consequences or alternative treatments [3, 5]. The principle of informed consent is based on the human right for self-determination and the ethical principle of autonomy [2, 6]. Informed consent is not only required by ethical aspects, but also incorporated in legal requirements. These legal requirements vary between countries [1–3]. The Council of Europe’s Convention on Human Rights and Biomedicine (chapter II, article 5), for example, declares that ‘an intervention in the health field may only be carried out after the person concerned has given free and informed consent to it. This person shall beforehand be given appropriate information as to the purpose and nature of the intervention as well as on its consequences and risk.’ For example in Germany, the role of patients has been strengthened by the Patient Rights Act in 2013 (Patientenrechtegesetz), that is incorporated into the Civil Code (para. 630a-630h BGB). Although, verbal consent is sufficient, practitioners in Germany frequently use informed consent forms to support the physician-patient-communication and to document written informed consent [5]. In contrast, the U.S. American law requires written informed consent as a prerequisite for interfering with the patient’s physical integrity [1].

This study aims at investigating the effects of evidence-based informed consent forms for total knee arthroplasty (TKA) surgery (including related anesthesia procedures) in clinical practice. Usually, informed consent for surgery does not primarily aim at proving adequate information to support an informed decision, because the decision for or against the medical intervention is usually made before hospital admission, e.g., during first consultation in primary care. However, it is not ensured that relevant information for the decision is provided in these consultations. Due to high liability risks for treatment errors, informed consent for surgery and related consent forms often focus on risk-centered information [3, 4, 7]. The way of presenting treatment risks can affect patients’ anxiety and the nocebo effect, which can be defined as ‘unpleasant or adverse outcomes triggered by the treatment context, beyond any inherent […] effects of the treatment itself’ [8–11]. Previous research shows that supporting documents (e.g., informed consent forms) used in practice are heterogeneous [12, 13] and that they are often deficient regarding the communication of risks [13].

## Objectives

The overall aim of this project is to investigate whether newly developed evidence-based informed consent forms for surgery and anesthesia (on the example of TKA) can reduce the deficits of the standardized consent forms regarding patients’ anxiety, nocebo effects, and risk perception. For this purpose, we will develop evidence-based informed consent forms and compare them with standard consent forms as used in routine care in Germany.

Specifically, we will perform the following sub-studies:

I. Qualitatively explore the information acquisition process and the decision-making processes
II. Develop (including pilot testing) evidence-based informed consent forms for total knee arthroplasty and related anesthesia procedures (regional and general anesthesia)
III. Conduct an interrupted time series (ITS) pilot study to test the effects of evidence-based informed consent forms in comparison with standard consent forms
IV. Perform a formative process evaluation to identify barriers and facilitators to the implementation of the intervention and to analyze contextual factors and mechanisms of impact

## Logic model

**Fig. 1:**
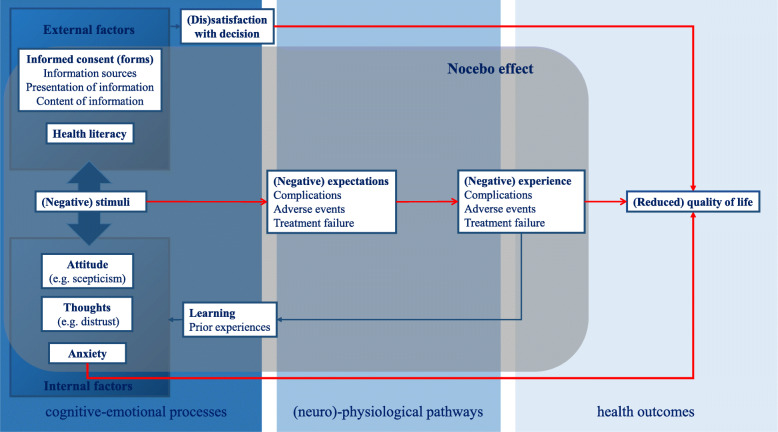
Logic model

The potential impact of information presented in informed consent forms on health outcomes depends on complex processes. Figure 1 depicts these complex processes and how informed consent forms may affect health outcomes.

Negative expectations are important elements of the complex processes which may produce nocebo effects [11]. Negative expectations are caused by negative stimuli, which are the results of assimilating internal and external factors through complex cognitive-emotional-processes [14]. External factors are primarily the information from informed consent forms. Other important factors might be pre-existing health literacy and other information sources. The source and content of information and the presentation of information may influence which subjective meaning is given to the information. Assimilating these external factors can evoke internal factors, in particular emotions like fear or even anxiety [14].

Via (neuro-)physiological pathways negative expectations, caused by the negative stimuli, may influence the likelihood of experiencing these expectations (e.g., headaches). This may in turn negatively affect the quality of life [15, 16].

In addition, anxiety [17] and dissatisfaction with the decision may have an immediate negative impact on quality of life.

## Methods

**Fig. 2:**
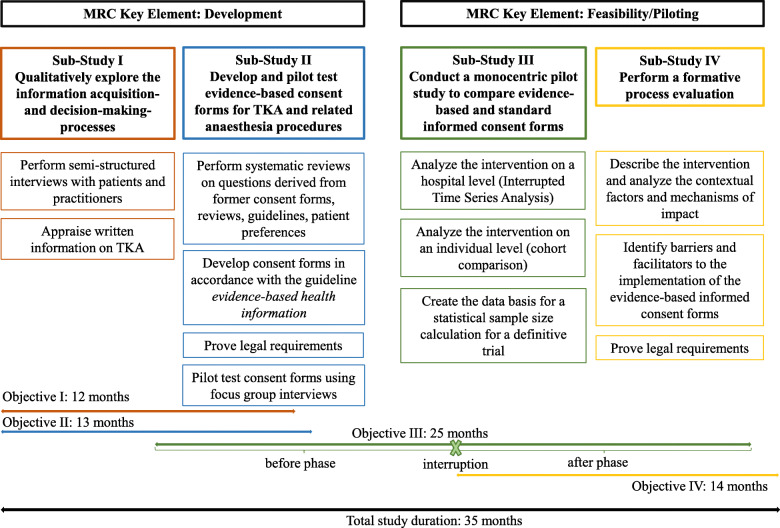
Sub-studies in accordance with the UK MRC Framework

The development, piloting, and evaluation of evidence-based informed consent forms for TKA and related anesthesia procedures will follow the UK MRC Framework for developing and evaluating complex interventions [18]. The UK MRC Framework describes four key elements of the development and evaluation process: (1) development, (2) feasibility/piloting, (3) evaluation, and (4) implementation. This study will include the first two key elements: Sub-studies I and II will be performed for developing the intervention; sub-studies III and IV will be performed for exploring feasibility and pilot testing the intervention (see Fig. 2).

This study protocol is reported according to the *Standard Protocol Items: Recommendations for Interventional Trials (SPIRIT)* [19], where applicable.

The study—including all sub-studies—is approved by the Ethics committee of the Witten/Herdecke University (reference number 198/2020). We will receive written informed consent from all participants prior to inclusion.

## Development

We will perform sub-studies I and II to develop the intervention.

### Sub-study I: exploration of the information acquisition process and the decision-making processes

We will explore the information acquisition processes and the decision-making processes to better understand the context in which the informed consent form is embedded. The informed consent on TKA and anesthesia is the final step in the decision-making process. Our aim is to describe the whole process to understand when, how, and by whom decisions are made and what information is decisive. We will derive information needs and preferences of patients, and identify barriers and facilitators for the adequate use of evidence based informed consent forms in practice.

We will perform semi-structured interviews with patients and physicians. Patients will be asked to describe their personal course of knee osteoarthritis. For specific situations in the course, they will be asked open-ended questions with regard to the objective. For example: how do you describe the information you received? How did your decision to have the surgery come about? Physicians, for example, will be asked about the information they give patients and how they involve patients in decision making. Patients, ≥ 18 years, with decision-making capacity, considering TKA or with previous TKA in the last 6 months will be included. Physicians (orthopedics and anesthetists) obtaining informed consent in the clinic for orthopedics, trauma surgery, and sports traumatology of the Cologne-Merheim Hospital as well as referring orthopedics and primary care physicians are eligible. Physicians in the clinic will be personally addressed, and referring physicians will be invited by email and phone. If they agree to participate, they will also receive information flyer for patients. Patients will be recruited by study nurses or physicians during the office hours of the clinic or ambulant consultations. Written informed consent will be obtained. Due to the corona pandemic, we will conduct online or telephone interviews. The interviews will be audio recorded and transcribed. We will perform a qualitative content analysis to develop categories and report on commonalities and differences. The analysis will be performed in an iterative process until no new categories can be derived and data saturation is achieved [20].

In addition, written information (leaflets, decision aids, and consent forms) on TKA provided by participating physicians or received by included patients, will be collected and rated with the ensuring quality information for patients (EQIP) scale [21] regarding criteria of evidence-based health information. Descriptive analysis will be performed.

### Sub-study II: development and pilot testing of evidence-based informed consent forms

#### Development of evidence-based informed consent forms

To promote informed choices, evidence-based informed consent forms on TKA and related anesthesia procedures (regional and general anesthesia) will be developed, taking insights from sub-studies I and II into account [22–26].

The design of the evidence-based informed consent forms, will be guided by an existing guideline on how to present evidence-based health information (*guideline evidence-based health information* [27]) and nocebo research. The information will be presented in plain language. Benefits and harms of the procedures will be presented in absolute risk formats and in comparison with other interventions or placebo [28]. Verbal presentations of risks lead to overestimations of risks [29] and therefore, will not be applied exclusively. Gain and loss framing will be combined. To visualize important aspects, pictograms will be used. Information on uncertainty, missing, or low-quality evidence will be provided.

We will perform systematic reviews on the comparative effectiveness and harms of TKA and related anesthetic procedures to generate quantitative information for developing the informed consent forms. Relevant research questions for the systematic reviews will be derived from former consent forms, reviews, guidelines, patient preferences, and in cooperation with the participating practitioners. We will include systematic reviews, randomized controlled trials, and prospective cohort studies. For rare adverse events and complications, we will consider database-based studies such as medical device registries or adverse event reporting systems, in addition. The quality of evidence will be rated according to GRADE [30]. Eligibility-screening of references, data extraction, and critical appraisal will be performed by two independent reviewers.

To resume the current state of research, we will review comparative studies on written material to promote informed consent identified in previous systematic reviews on this topic [31, 32].

We will derive relevant information on benefits and risks for the knowledge test, for usage in the main study (objective III). The development will be based on previous experiences with the measurement of informed choice [33, 34].

Due to the special legal requirements for informed consent, we will prove evidence-based consent forms for satisfying legal requirements. The lawyers of our team will perform a comprehensive evaluation of case law and literature (monographs, articles, commentaries, especially on the §§ 630d, 630e BGB) on the legal aspects regarding informed consent in Germany. During this process, the legal scope that may result from more recent jurisdiction on the physician’s obligation to provide information and the requirements for evidence-based informed consent forms will be examined and compared. At the same time, the legal framework for future patient-oriented education is defined in the concept of informed consent.

#### Pilot testing

The new consent forms will be tested on comprehensibility, readability, acceptance, and emotional response. Think-aloud interviews are planned, in which participants share their thoughts while reading the consent form, followed by at least two focus group interviews with patients of the target group. The interviews will be audio recorded and transcribed. Qualitative content analyses will be performed [20]. The consent forms will be revised and tested in an iterative process until data saturation is achieved.

The knowledge questions for sub-study III will also be pilot tested. We will optimize the items and select the most appropriate for the knowledge test.

In addition, a member from the *Health Information Department* of the independent *Institute for Quality and Efficiency in Health Care* (IQWiG), clinical experts and lawyers from the respective scientific medical societies (*The German Society for Endoprosthetics* and *The German Society for Anaestesiology and Intensive Care Medicine*, *DGAI*) and the study center will review the informed consent forms.

## Feasibility/piloting

### Sub-study III: monocentric interrupted time series (ITS) pilot study

We will conduct a monocentric pilot study. This pilot study will be performed following a pragmatic trial approach to increase applicability to the German context of informed consent procedures [35]. Our primary analysis is an ITS analysis approach. Using an ITS analysis, will allow us to compare the outcome trend (level and/or slope changes) before and after the introduction of the newly developed evidence-based informed consent forms [36].

### Methods: participants, interventions, and outcomes

#### Study setting

The study will be conducted in an urban region (Cologne) at a level III hospital in Germany (Cologne-Merheim Hospital).

#### Eligibility criteria

Participants eligible for inclusion in the study:

- Are scheduled for an elective total knee arthroplasty surgery
- Are at least 18 years old
- Are able to understand and speak German
- Are mentally competent to give consent and answer questions

Patients with revision total knee arthroplasty (rTKA) or post-traumatic arthrosis will be excluded.

Hospital staff or study nurses will check the inclusion criteria for each potentially relevant patient at consultation during the office hours of the clinic for orthopedics, trauma surgery and sports traumatology of the Cologne-Merheim Hospital.

#### Interventions

We include two groups of participants before (pre) and after (post) the introduction of the intervention. The group in the pre period (control group) will give informed consent based on standard consent forms from the Thieme-compliance publisher (https://shop.thieme-compliance.de/patientenaufklaerung/thieme/de/Artikel/Aufkl%C3%A4rungsb%C3%B6gen/c/00000), which represents routine care in the study center. The group in the post-period (intervention group) will give informed consent based on our newly developed evidence-based informed consent forms (see sub-study II). The change of the informed consent forms will be introduced at a pre-specified time-point. The informed consent procedures for surgery and for anesthesia will be performed together as far as feasible in the usual hospital workflow.

In addition, physicians performing informed consent in the post-phase will be offered a training in evidence-based decision-making to adequately implement the evidence-based informed consent forms. The training will be based on the basic curriculum for evidence-based decision-making, developed by the *German Network for Evidence-based Medicine*. A curriculum-based, blended learning training program for physicians and medical students has already been developed and pilot tested [37]. This training will be converted to an e-learning module to be independent of possible restriction due to the COVID-19 pandemic. In addition, e-learning with only short phases of virtual presence offers more flexibility. The training will also be adapted to address evidence-based decision-making on TKA. For major changes pilot testing will be considered.

#### Blinding

Standard consent forms and evidence-based informed consent forms are obviously different. Therefore, blinding of medical staff will be impossible. We will not explicitly inform participants about receiving a “different” informed consent form but only about the nature of the study. The statistical analysis will be performed blinded.

#### Outcomes and measurement instruments

We plan to measure the following patient-relevant outcomes:

- Primary outcomes:
- Anxiety: subjective fear/anxiety of adverse events possibly caused by surgery or anesthesia
  - ○ Measuring instrument: numeric rating scale 0–10 (validation see [38, 39]) and surgical fear questionnaire (SFQ) [40]. The SFQ comprises 8 items reflecting different aspects of surgery-related fear (e.g., anesthesia or side effects). All items are scored on a numeric rating scale 0–10. It is validated in adult patients scheduled for an elective surgery.
  - ○ Measure: mean
- Nocebo-effect: patient reported adverse events (e.g., headache after anesthesia)
  - ○ Measuring instrument: a questionnaire will be developed. We will use closed simple-choice questions (yes/no) to detect frequent adverse events and open-ended questions to detect other (less common) adverse events.
  - ○ Measure: proportion of patients with complications or adverse events
- Secondary outcomes:
- Knowledge or risk perception: questions for knowledge about benefits and risks
  - ○ Measuring instrument: objective knowledge questions (e.g., correct risk assessment) derived in the development phase. The development will be based on previous experiences with the measure of informed choice [33, 34].
  - ○ Measure: proportion of correctly answered questions
- Satisfaction with the physician-patient-communication and informed consent form
  - ○ Measuring instrument: numeric rating scale 0–10 (validation see [41])
  - ○ Measure: mean
- Quality of life (QoL)
  - ○ Measuring instrument: numeric rating scale 0–100 (validation see [42])
  - ○ Measure: mean
- Pain and function
  - ○ Measuring instrument: Oxford Knee Score (OKS) [43]. The OKS is a 12-item questionnaire with five scoring categories (validation see [43]).
  - ○ Measure: mean

#### Sample size

To our knowledge, no comparative studies on different informed consent forms for TKA and related anesthesia procedures in the German context exist so far. Therefore, a sufficient data basis for statistical sample size calculation is not available. With this pilot study, we aim to create the data basis for sample size calculation and detect unforeseen problems (e.g., in recruitment, processes) for a definitive trial to prove effectiveness. We calculated the sample size for this pilot study using the probability to identify unforeseen problems [44] as anticipated to provide a robust basis for a valid sample size calculation in a definitive trial.

We plan to include at least 220 participations (110 pre, 110 post) in the study. Based on findings from previous studies [10], we anticipate a dropout rate of 10% so that 198 participants can be included in the analysis. Using this sample size, unforeseen problems with a 2% probability of occurrence can be identified with a probability of 98%. We based the sample size calculation on such conservative values to increase the planning certainty of a future trial.

#### Recruitment

Based on the yearly caseload of patients with TKA in the study center, we anticipate a recruitment phase of 24 months. In the first 12 months participants receive the standard consent form (12 months pre-phase). After this period, the evidence-based consent form will be introduced and subsequent participants will receive the evidence-based consent form (12 months post-phase). Recruitment is expected to start in March 2021 and finish in February 2023. Study participants will be recruited by study nurses or physicians during the office hours of the clinic for orthopedics, trauma surgery, and sports traumatology of the Cologne-Merheim hospital. There will be no financial incentives for participation.

### Participant timeline

Table 1 depicts the participant timeline. The study includes a pre-interruption and post-interruption-phase. Participants in the pre-phase will receive informed consent based on standard consent forms (control) while participants in the post-phase will receive informed consent based on the newly developed evidence-based consent forms (intervention). Irrespective of the phase, all participating patients will follow the same study flow: study enrollment of each patient will take place approximately 4 weeks before surgery. They will receive informed consent procedures for TKA approximately 2–4 days before surgery. The informed consent procedure for anesthesia takes place approximately 4 weeks or 2–4 days before surgery, depending on the patients’ morbidity and the flexibility in scheduling. Outcomes will be measured at four specific time points for each patient: at the day of enrollment, 1 day before surgery, 3 days and 30 days after discharge from hospital.

**Table 1:** Participant timeline (SPIRIT figure)

	Study period (25 months)													
		Pre-phase						Interruption	Post-phase					
	Time-point	4 weeks before surgery	2–4 days before surgery	1 day before surgery	Surgery	3 days after discharge	30 days after discharge		4 weeks before surgery	2–4 day before surgery	1 day before surgery	Surgery	3 days after discharge	30 days after discharge
**Enrolment**	**Eligibility screen**	X							X					
	**Informed consent**	X							X					
**Control**	**Standard consent form anesthesia**	X^a^	X^a^											
	**Standard consent form TKA**		X											
**Intervention**	**Evidence-based consent form anesthesia**								X^a^	X^a^				
	**Evidence-based consent form TKA**									X				
	**Surgery**				X							X		
**Assessments**	**Patient characteristics**	X							X					
	**Baseline anxiety**	X							X					
	**Anxiety**			X							X			
	**Health knowledge**			X							X			
	**Satisfaction with physician-patient-communication**			X							X			
	**Nocebo-effect**					X							X	
	**Quality of Life**						X							X
	**Pain and function**						X							X

^a^Informed consent for anesthesia takes place approximately 4 weeks or 2–4 days before surgery, depending on the patients’ morbidity and the flexibility in scheduling

### Methods: data collection, management, and analysis

#### Data collection and management

We will collect the participants’ characteristics (e.g., education) using patient surveys as standardized in German socio economic panels [45]. All outcomes will be collected with standardized patient surveys. All patient surveys will be performed by the same four interviewers based on an interviewer manual to ensure consistency.

Data will be collected using an electronic case report form (eCRF) and stored in an electronic data management system (REDCap) [46, 47]. The eCRF and data management system will be piloted with dummy participants (hypothetical patients). We will perform a sample of double data entry to prove data quality. Only authorized study personal will have access to the data management system.

To ensure high retention, the participants’ expenditure of time for study participation will be kept as low as possible. Moreover, due to the nature of the intervention, there will be no physical strain for the participants caused by study participation.

#### Statistical model

We will report baseline characteristics of the study population and outcomes descriptively.

We will use an ITS analysis to determine level and slope changes after the implementation of the evidence-based informed consent forms. We will use segmented regression models for our analysis. The basic model is *Y*_*t*_ = *β*_0_ + *β*_1_*T*_*t*_ + *β*_2_*X*_*t*_ + *β*_3_(*T* − *T*_0_)*X*_*t*_

*X*_t_ is a dummy which differentiates pre- and post-intervention periods. *T* is the time since the start of the study in months. β_0_ is the base level. β_1_ is the course of the slope before the implementation of the intervention. β_2_ is the level change, which describes the immediate effect on *Y* after the implementation of intervention *X*. β_3_ is the course of the slope after the implementation of the intervention, whereby *T*_o_ is the time of beginning of the study. We will use negative-binomial regression models for all count data (nocebo effect and objective knowledge questions) and generalized linear model for outcomes which can be analyzed as continuous variables (anxiety, satisfaction, quality of life, OKS).

We will create two models. One model will analyze the intervention as a complex package of measures on hospital level. The intervention effect will be analyzed on the hospital level. We will adjust the analysis for potential unforeseen time-varying confounders (e.g., changes of the standard consent form by hospital information policies, structural changes in consent because of corona pandemic). Potential confounders for each outcome will be identified based on visual inspection of trajectory curve and content-related thinking. We will assess autocorrelation by examining the plot of residuals.

In a second model, we will analyze the intervention effect on an individual level. This means, the study is analyzed as historical cohort study. In this model, the individual intervention components (informed consent forms, parallel performance of informed consent by orthopedic and aesthetic medical staff, participation on the training for evidence-based decision-making) are modelled as separate intervention components. We will perform this exploratory analysis to get insights into the isolated effect of evidence-based informed consent forms and interaction effects with other intervention components. We will adjust the models for baseline characteristics (age, sex, educational level, and marital status) and a potential time trend (since the comparison group is a historical control).

In case > 5% of data for an outcome are missing, we will impute the missing data by multiple imputation using the Markov chain Monte Carlo method. In addition to this primary analysis, we will perform a best-case/worst-case analysis to assess the robustness of results depending on missing data [48].

To quantify the statistical uncertainty, the associated 95% confidence intervals will be provided for all effect estimates. As we will conduct an explorative pilot study, we will not perform a test for statistically significant differences. We will prepare interrupted-time-series graphs to visualize the results [49].

All analyses will be performed using SAS software [50] and R [51].

#### Methods: monitoring

Due to the nature of the intervention, there will be no data monitoring committee.

#### Sub-study IV: process evaluation

Following the Medical Research Councils process evaluation framework for complex interventions [52], we will plan and conduct the process evaluation. The detailed planning will be based on the results of the sub-studies I and II. We will describe the intervention, its casual assumptions and the implementation process. We will analyze the contextual factors and the mechanisms of impact such as participant responses and mediators. The legal framework of informed consent will always be considered. Mixed methods will be applied to assess intervention fidelity and the processes and mechanisms on different levels. The informed consent process will be documented on a standardized form (e.g., duration, orthopedist and anesthetist together, new informed consent form used yes/no, consent provided yes/no). The context will be explored using the Practice Adaptive Reserve Scale [53]. The model of adaptive reserve considers external factors as well as leadership, learning culture, teamwork, or characteristics of the relationships in the work environment. Readiness to change on a healthcare professional level will be surveyed using the German Version of the Change Attitude Scale [54]. The Change Attitude Scale is a three-dimensional tool, measuring the affective (how people feel about the change), cognitive (what people think about the change), and behavioral (behavior in response to the change) dimension. Barriers and facilitators of implementation will be explored using qualitative methods. We will perform semi-structured interviews with patients and physicians. Participating orthopedists and anesthetists who regularly perform informed consent procedures and a random sample of patients in the intervention group (20% of participants) will be included. The selected patients will be contacted by telephone approximately 1 month after informed consent. At the end of the study, personal interviews with the physicians will be scheduled if possible. Otherwise, they will also be contacted by telephone. The interviews will be audio recorded and transcribed. Qualitative content analyses will be performed [20]. A descriptive analysis will be performed on the quantitative data.

## Discussion and practical implications

The evidence-based and understandable presentation of risks in informed consent forms aims at avoiding distorted risk depiction and strengthening the patients’ competence to correctly assess the risks of undergoing surgery. This might reduce negative expectations and anxiety of negative consequences, which in turn might reduce the nocebo effect. At the same time, the practitioners’ acceptance of evidence-based informed consent forms meeting legal requirements may be increased.

Studies on informed consent forms indicate that the presentation of benefits and harms might impact patients’ pre-operative anxiety and competence to correctly assess treatment risks [55, 56]. These studies primarily examine personalized information options (e.g., IT-based) and supplemental information materials in the context of shared decision-making. To the authors’ knowledge, there are no studies comparing different standardized, paper-based informed consent forms. Moreover, studies on risk communication show that the way in which treatment risks are presented can have an impact on patients’ anxiety and the nocebo effect [9, 28, 57]. However, these studies are artificial, because they either examined only isolated aspects (e.g., illustrated versus verbal description of risks) or were conducted under highly experimental circumstances (e.g., verbal suggestion to an artificial negative stimulus). Therefore, the generalizability of the results to routine care is questionable. In the German context, a randomized controlled pilot study was conducted which compared a modified package leaflet (developed according to the guideline for evidence-based health information [27]) with a standard (EU regulation-compliant) package leaflet for ibuprofen. The results indicate that these modifications can reduce anxiety and nocebo effects while still increasing patients’ comprehension and knowledge [10].

This study will be performed under routine conditions. Despite focusing on only one indication, we anticipate that the basic impact tendency may be generalized to other indications and settings. If this pilot study indicates positive effects of the newly developed evidence-based informed consent forms, it is planned to perform a definitive cluster-randomized trial for a wide range of different indications. Relations to publishers of informed consent forms already exist. Thus, if this pilot study indicates positive effects of evidence-based informed consent forms, this is a good starting situation to develop further evidence-based informed consent forms in cooperation. The use of informed consent forms is widespread. Therefore—premised a definitive study shows that the improvement of patient-relevant outcomes is possible—a large-scale implementation could have an important positive impact on a population level.

## Limitations

The study was planned as a non-randomized study and is therefore at risk for confounding bias. Moreover, this is a time series study which might be biased by temporal effects. The risk of temporal changes is in particular high because of the dynamic COVID-19 pandemic (e.g., hospital limits surgery to patients with acute pain). A randomized controlled trial would control for unmeasured confounding. However, we decided against a randomization on individual level because we anticipated that there is a high risk of bias due to contamination. The study was not planned as a cluster-randomized trial because this would have exaggerated the expense for a pilot-trial.

Finally, external validity might be limited because the study is only performed in one hospital in Germany.

## Data Availability

Anonymized data of the pilot-study will be published at Open Science Framework https://osf.io/.

## Abbreviations

BGB: Bürgerliches Gesetzbuch (German Civil Code)
DGAI: Deutsche Gesellschaft für Anästhesiologie und Intensivmedizin (German Society for Anaestesiology and Intensive Care Medicine)
DNEbM: Deutsches Netzwerk Evidenzbasierte Medizin (German Network for Evidence-based Medicine)
eCRF: Electronic Case Report Form
G-BA: Gemeinsamer Bundesausschuss (German Federal Joint Committee)
ITS: Interrupted time series
IQWiG: Institut für Qualität und Wirtschaftlichkeit im Gesundheitswesen (Institute for Quality and Efficiency in Health Care)
QoL: Quality of life
OKS: Oxford Knee Score
REDCap: Research Electronic Data Capture
rTKA: Revision Total Knee Arthroplasty
SFQ: Surgical Fear Questionnaire
SPIRIT: Standard Protocol Items: Recommended for Interventional Trials
TKA: Total knee arthroplasty
UK MRC: United Kingdom Medical Research Council
